# Supplementary material for: Dynalign II: common secondary structure prediction for RNA homologs with domain insertions
Source: Nucleic Acids Res. 2014 Nov 21;42(22):13939–48. doi: 10.1093/nar/gku1172 (PMC4267632; doi:10.1093/nar/gku1172)
Supplement: SUPPLEMENTARY DATA [file supp_gku1172_nar-02021-z-2014-File012.zip › manual/Text/AlphabeticalIndex.html]

RNAstructure Command Line Help: Index


|  |  |  |
| --- | --- | --- |
|  | RNAstructure Command Line Help Index | - Contents - Index |
| A B C D E F G H I J K L M N O P Q R S T U V W X Y Z --- A Alignment File Format AllSub: Overview AllSub: References AllSub: Run Information \| N NAPSS: Overview NAPSS: References NAPSS: Run Information NMR Data Integrated With Structure Prediction; B bifold: Overview bifold: References bifold: Run Information Bimolecular Folding Bimolecular Duplex Folding Bimolecular Partition Function bipartition: Overview bipartition: References bipartition: Run Information Building the Text Interfaces \| O Offset File Format oligoscreen: Overview oligoscreen: References oligoscreen: Run Information OligoWalk; C Calculating Ensemble Energy Calculating Common Structures For Multiple Sequences Calculating Common Structures For Two Sequences CircleCompare: Overview CircleCompare: References CircleCompare: Run Information Comparing Two Structures Converting Bracket Files to CT Files Converting CT Files to Bracket Files CT File Format ct2dot: Overview ct2dot: References ct2dot: Run Information cuda: partition-cuda \| P partition: Overview partition: References partition: Run Information Partition Function Single Strand PARTS: Overview PARTS: References PARTS: Run Information Probability of Pairs in Two Unaligned Sequences ProbabilityPlot: Overview ProbabilityPlot: References ProbabilityPlot: Run Information ProbablePair: Overview ProbablePair: References ProbablePair: Run Information ProbKnot: Overview ProbKnot: References ProbKnot: Run Information Pseudoknot Prediction: ProbKnot Pseudoknot Prediction: ShapeKnots; D dot2ct: Overview dot2ct: References dot2ct: Run Information draw: Overview draw: References draw: Run Information Drawing Secondary Structures Drawing a Base Pairing Probability Dot Plot Drawing a Dynalign Dot Plot Drawing a Folding Free Energy Dot Plot DuplexFold: Overview DuplexFold: References DuplexFold: Run Information dynalign: Overview dynalign: References dynalign: Run Information dynalign-smp: Overview dynalign-smp: References dynalign-smp: Run Information DynalignDotPlot: Overview DynalignDotPlot: References DynalignDotPlot: Run Information \| Q; E efn2: Overview efn2: References efn2: Run Information Energy Function 2 EnergyPlot: Overview EnergyPlot: References EnergyPlot: Run Information EnsembleEnergy: Overview EnsembleEnergy: References EnsembleEnergy: Run Information Experimental Pair Bonus File Format \| R refold: Overview refold: References refold: Run Information Refolding Previously Folded Structures RemovePseudoknots: Overview RemovePseudoknots: References RemovePseudoknots: Run Information Removing Pseudoknots; F FASTA File Format File Formats Folding Free Energy Calculation Fold: Overview Fold: References Fold: Run Information Folding Constraint File Format Folding Single Stranded Structures \| S scorer: Overview scorer: References scorer: Run Information Scoring a Structure's Quality Sequence File Format SHAPE Restraint File Format ShapeKnots Specifying Thermodynamic Parameters Stochastic Sampling; G Generating All Suboptimal Structures Generating Probable Structures \| T Thermodynamic Parameters for Oligos TurboFold: Overview TurboFold: References TurboFold: Run Information; H \| U; I Identifying Pseudoknots \| V; J \| W; K \| X; L List File Format \| Y; M MaxExpect: Overview MaxExpect: References MaxExpect: Run Information Maximum Expected Accuracy Structures Multifind: Overview Multifind: Run Information multilign: Overview multilign: References multilign: Run Information multilign-smp: Overview multilign-smp: References multilign-smp: Run Information \| Z | | |
| Visit The Mathews Lab RNAstructure Page for updates and latest information. | | |
